# A case report of ruptured inferior mesenteric artery aneurysm with superior mesenteric artery stenosis

**DOI:** 10.3389/fcvm.2026.1790613

**Published:** 2026-03-26

**Authors:** Long Guo, Meili Lu, Zhiliang He

**Affiliations:** 1 Department of Intensive Care, Qilu Hospital of Shandong University Dezhou Hospital (Dezhou People's Hospital), Dezhou, Shandong, China; 2 Department of General Practice, Qilu Hospital of Shandong University Dezhou Hospital (Dezhou People's Hospital), Dezhou, Shandong, China; 3 Department of Radiology, Qilu Hospital of Shandong University Dezhou Hospital (Dezhou People's Hospital), Dezhou, Shandong, China

**Keywords:** diagnostic angiography, inferior mesenteric artery aneurysms, personalized treatment strategy, superior mesenteric artery stenosis, transcatheter arterial embolization (TAE)

## Abstract

The incidence of inferior mesenteric artery aneurysms is extremely low. Once they rupture, the situation becomes extremely dangerous, and immediate intervention measures must be taken. We reported a unique case of ruptured and bleeding inferior mesenteric artery aneurysm with superior mesenteric artery stenosis. After diagnostic angiography, a super-selective transcatheter arterial embolization (TAE) was performed. Our patient achieved the best therapeutic outcome through the most minimally invasive surgical method. A personalized treatment strategy is recommended in the management of inferior mesenteric artery aneurysm.

## Introduction

An aneurysm refers to permanent, focal bulging of an arterial wall that results in the vessel's diameter being more than 50% larger than its normal size. An inferior mesenteric artery (IMA) aneurysm is considered one of the splanchnic artery aneurysms, accounting for approximately 1% of visceral artery aneurysms (1, 2). This is an extremely dangerous and urgent condition that can lead to fatal intra-abdominal hemorrhage and intestinal ischemia, requiring urgent intervention. The objective of this study was to report a rare case of an inferior mesenteric artery aneurysm with superior mesenteric artery stenosis, which was treated by arterial embolization, and to describe the long-term outcome. Written informed consent for publication of the case and accompanying images was obtained from the patient.

## Case description

**Figure 1:**
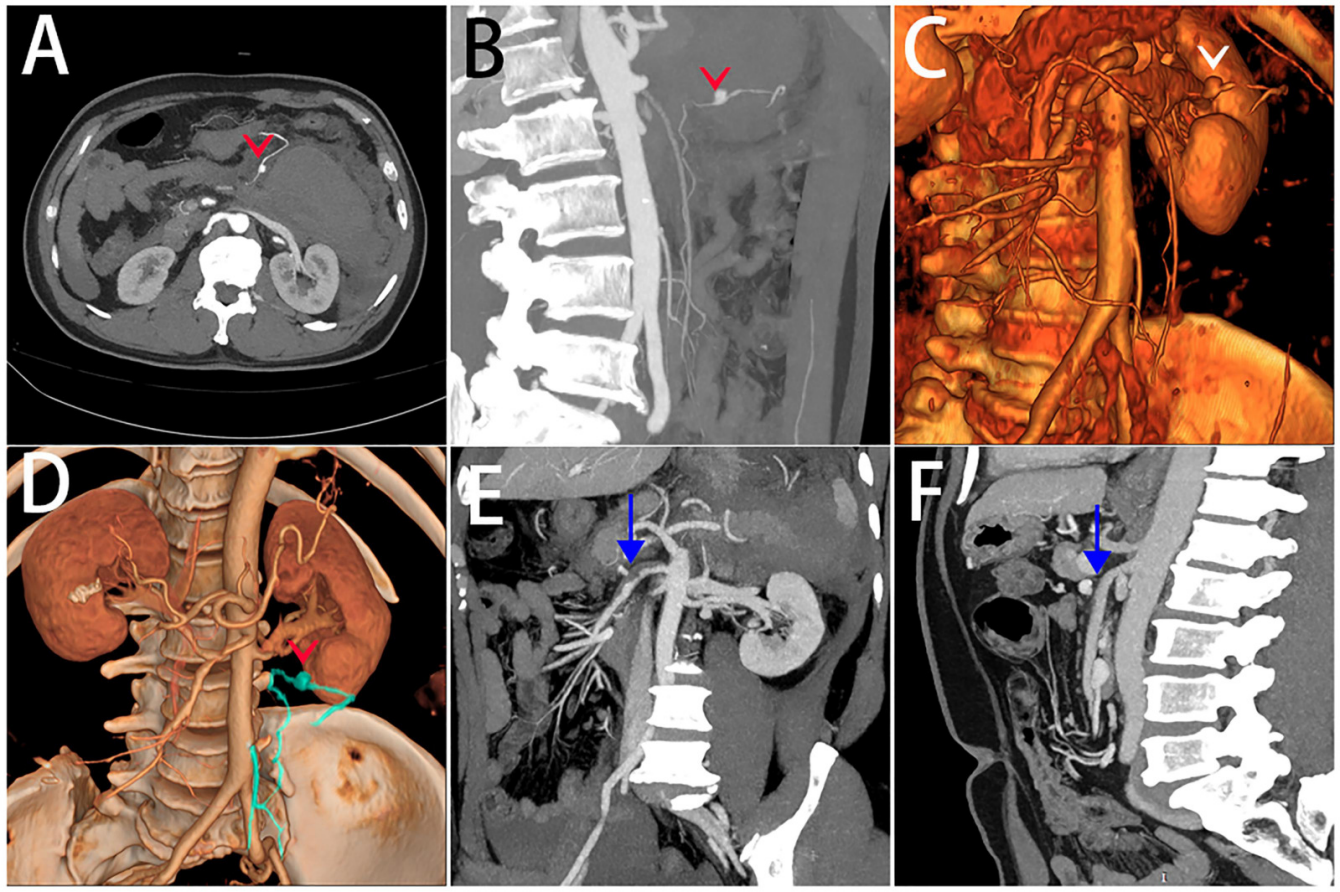
Images in a 57-year-old man with an inferior mesenteric artery aneurysm. **(A–D)** CT shows abdominal hematoma in the left upper quadrant, and cinematic renderings from CT angiography show a small aneurysm of the inferior mesenteric artery (v). Intestinal artery CTA shows moderate stenosis of the proximal superior mesenteric artery **(E**↓**)** and no significant stenosis of the superior mesenteric artery **(F**↓**)**.

A 57-year-old man was admitted to the hospital with a 1-day history of abdominal pain and a half-day history of unconsciousness. He had been previously healthy, with no history of hypertension, diabetes, major trauma, or abdominal surgery. On physical examination, the patient appeared anemic but was hemodynamically stable; however, he exhibited abdominal tenderness in the mid to upper abdomen. Laboratory test revealed a hemoglobin level of 101 g/L. Abdominal CT showed the presence of a hematoma. Whole abdominal computed tomography angiography (CTA) demonstrated an inferior mesenteric artery aneurysm (Figures 1A–D) and moderate stenosis of the proximal superior mesenteric artery (Figure 1E). Following a multidisciplinary discussion, it was unanimously agreed that the hematoma formation was caused by rupture of the inferior mesenteric artery aneurysm. Digital subtraction angiography (DSA)-guided selective arteriography of the inferior mesenteric artery was performed under local anesthesia, when the patient's vital signs were stable.

**Figure 2:**
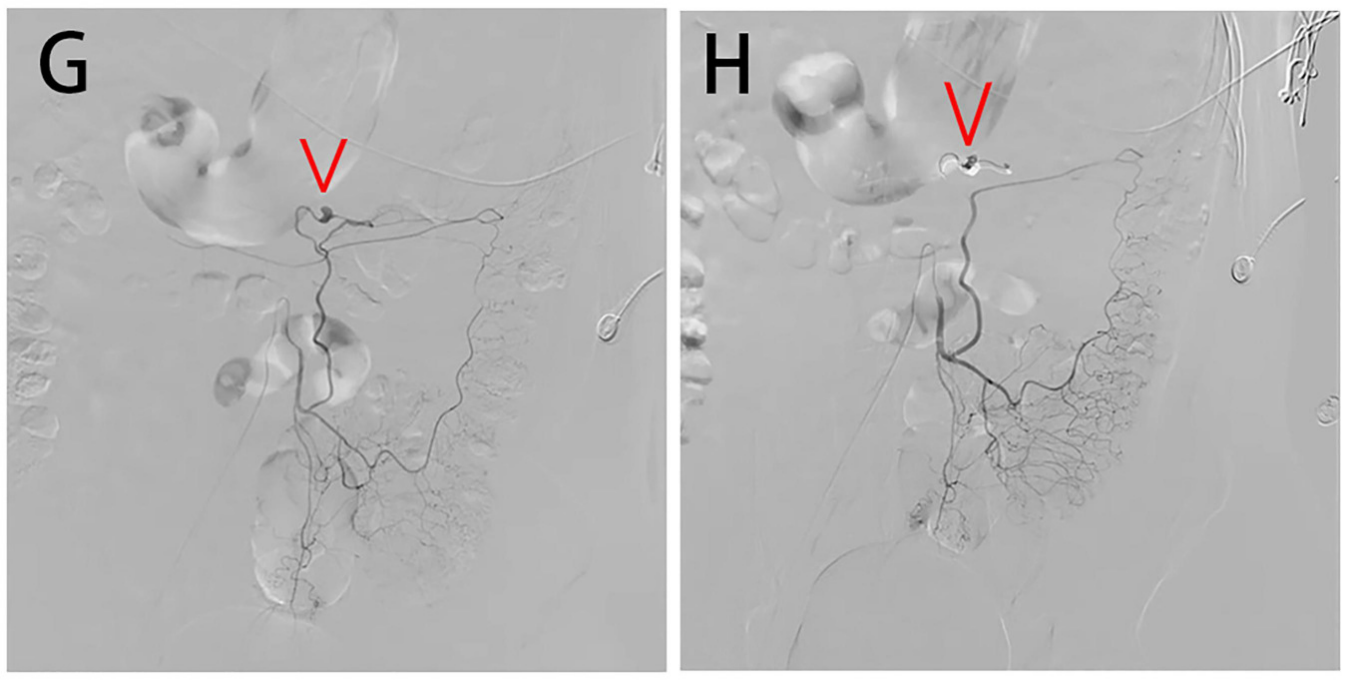
**(G)** Angiography showing the inferior mesenteric artery aneurysm (IMA). **(H)** Final angiography showing complete occlusion of the aneurysm lumen of the IMA.

Inferior mesenteric artery angiography revealed a 1.0 cm aneurysm at the distal end of the left colic artery (a branch of the inferior mesenteric artery), with irregular margins (Figure 2G). A guidewire and catheter were used to navigate the diseased artery, and the aneurysm was embolized with pushable coils at both the proximal and distal ends (Figure 2, Movies H); the patient's vital signs remained stable throughout the procedure. Repeat contrast angiography showed that the mesenteric artery branch aneurysms were no longer visualized, and there was approximately 70% stenosis at the proximal end of the superior mesenteric artery. Three days postoperatively, CT reexamination demonstrated that the abdominal hematoma had decreased in size compared with before, with no signs of intestinal necrosis. The patient was discharged eight days after the intervention, and no recurrence was observed at the 2-week, 1-month, and 3-month follow-ups. During the 6-month follow-up, the patient remained in good condition, and mesenteric artery CTA showed no significant stenosis of the superior mesenteric artery (Figure 1F).

## Discussion and conclusion

The development of inferior mesenteric artery aneurysms involves several mechanisms. Causes include degenerative or inflammatory conditions, fibromuscular dysplasia, collagen vascular diseases, and inherited disorders (3). An additional proposed mechanism is occlusive disease in other mesenteric arteries (4–6), which can result in hemodynamic changes such as increased flow, turbulence, and wall stress, ultimately leading to progressive dilatation and aneurysm formation (7).

Submesenteric aneurysms are usually asymptomatic and are most commonly discovered during physical examinations or while investigating the causes of abdominal pain (8). They may present with various symptoms upon rupture, such as gastrointestinal bleeding, abdominal hemorrhage, or retroperitoneal hematoma. Abdominal radiographs may occasionally reveal a calcified aneurysm, but this finding has little diagnostic significance. CTA plays an important role in establishing a definitive diagnosis due to its noninvasive nature. Intra-arterial DSA remains the gold standard for diagnosis and treatment of submesenteric artery aneurysms, as it clearly delineates the relationship between the aneurysm and surrounding blood vessels.

Edogawa et al. suggested that inferior mesenteric artery aneurysms with a diameter greater than 2 cm at the proximal portion or 1 cm at the distal segment should be treated surgically, considering the risk of rupture (9). Skalický et al. found that the preferred treatment for ruptured pancreaticoduodenal arcade aneurysms was endovascular intervention using transarterial embolization, which was associated with lower morbidity and mortality compared to surgical intervention (10). Endovascular treatment strategies represent the first line of treatment for the majority of visceral arterial aneurysms, although open surgery continues to play a role in specific conditions (11). Various recognized treatment options for inferior mesenteric artery aneurysms include aneurysmectomy, bypass or vascular reconstruction, ligation, aneurysm suture, and embolization (12).

Endovascular repair has been regarded as the first-line treatment method in recent years owing to its minimally invasive nature and rapid recovery (13–15). The main endovascular techniques involve the placement of covered stents and aneurysm embolization. Covered stents are suitable for complex aneurysms such as giant and wide-neck aneurysms, particularly those located in large vessels without significant side branches. Long-term antiplatelet therapy is required postoperatively to prevent in-stent thrombosis, with a relatively low bleeding risk. Aneurysm coiling embolization is suitable for various types of aneurysms, especially narrow-neck aneurysms. The coils are placed entirely within the aneurysm lumen without affecting arterial blood flow, and long-term antiplatelet or anticoagulant therapy is generally not required after the procedure.

In this case, as the patient was relatively young, endovascular aneurysm embolization was considered more appropriate based on the location and morphology of the aneurysm, as well as the patient's personal preference. The patient did not receive oral anticoagulants or antiplatelet medications postoperatively. Compared with surgical procedures, endovascular treatment is often associated with a shorter hospital stay and lower cost; however, its drawbacks cannot be ignored. Endovascular treatment may cause damage to the vascular intima, which can lead to the formation of secondary hematomas. In severe cases, it may also result in limb ischemia.

In our case, following arterial embolization for a ruptured inferior mesenteric artery aneurysm, the stenosis of the superior mesenteric artery resolved. When a hematoma is located in the abdominal cavity near the superior mesenteric artery, it may compress the vessel, causing temporary narrowing of the lumen and resulting in a “pseudo-stenosis”. This narrowing is not due to pathological changes of the vessel itself (such as atherosclerosis or dissection) but is caused by the external compression from the hematoma.

In conclusion, prompt diagnosis of mesenteric artery aneurysms should be emphasized. The treatment strategy should be flexibly chosen based on the clinical presentations, etiology of the aneurysm, anatomical characteristics of the lesion, perfusion status of the involved organs, and the presence of any concomitant diseases. Both endovascular treatment and open surgery have their own advantages and disadvantages. Individualized treatment is essential for achieving an optimal long-term prognosis.

## Data Availability

The datasets presented in this study can be found in online repositories. The names of the repository/repositories and accession number(s) can be found in the article/Supplementary Material.
